# Applications and outcomes of virtual reality in inpatient psychiatry: A systematic review

**DOI:** 10.1017/S0033291726105662

**Published:** 2026-09-03

**Authors:** Zachary Fontaine-Dussault, Lori Hazel, Logane Dupont, Clayton Jeffrey, Carolane Bernard-Gilbert, Florence Fontaine, Christina Soucy, Michelle Wang, Martin Lepage, Elisabeth Thibaudeau

**Affiliations:** 1 Universite de Montreal, Canada; 2 Universite Laval Faculte des sciences sociales, Canada; 3 Douglas Hospital Centre: Institut universitaire en sante mentale Douglas, Canada; 4 McGill University Department of Psychology, Canada

**Keywords:** inpatient, psychiatry, virtual reality

## Abstract

**Background:**

Virtual reality (VR) has been widely used in outpatient psychiatric services and has demonstrated benefits across several clinical diagnoses, but its use and effects in inpatient settings remain to be explored. This systematic review aimed to examine the use of VR during psychiatric hospitalization, including types of VR applications, barriers and facilitators of implementation, and effects on various outcomes.

**Methods:**

The review was registered in PROSPERO (#CRD42023446524). Following PRISMA guidelines, databases (Ovid, SciVerse, Web of Science, Cochrane Library, ProQuest, and WorldCat) were searched from 1983 to 2025 using keywords related to VR and psychiatric disorders. Studies involving the use of VR with psychiatric inpatients (≥85%) were included. Descriptive statistics and narrative syntheses were used to summarize findings. Study quality was assessed with the Mixed Methods Appraisal Tool.

**Results:**

After full-text screening, 37 studies (*N* = 1,004) met inclusion criteria. VR was used for both assessment and intervention, with cognitive-behavioral therapy/exposure (35%) and assessment (24%) being the most frequently used. VR use in inpatient units appeared feasible, acceptable, and safe for inpatients and clinicians, though findings remain preliminary. Several facilitators (e.g. adequate staff training and supervision) and common barriers (e.g. technical difficulties and limited resources) were identified. The most consistent improvements were observed in clinical symptoms (e.g. anxiety) compared with psychosocial, cognitive, and physiological outcomes.

**Conclusions:**

These findings suggest that inpatient settings represent a promising, yet understudied context for VR-based assessments and interventions. High-quality trials and systematic reporting of implementation are needed in future studies to inform research and clinical practice.

## Introduction

Virtual reality (VR) delivers immersive, multisensory environments via head-mounted displays, providing safe and controlled simulations that enhance engagement through interactive and gamified experiences. VR has demonstrated potential across diverse therapeutic applications in psychiatry (Freeman et al., 2017; Maples-Keller, Bunnell, Kim, & Rothbaum, 2017; Riva, Malighetti, & Serino, 2021). Previous systematic reviews and meta-analyses have reported that immersive VR interventions may improve clinical outcomes across psychiatric disorders and are generally feasible and acceptable (Carl et al., 2019; Fodor et al., 2018; Maples-Keller et al., 2017; Valmaggia, Latif, Kempton, & Rus-Calafell, 2016; Zeka et al., 2025). However, these reviews have primarily focused on outpatient populations or psychiatric disorders broadly, providing limited insight into the implementation, feasibility, and effectiveness of VR in psychiatric inpatient settings.

Although psychiatric inpatient care primarily emphasizes pharmacological stabilization (Alzahrani, 2021), psychosocial interventions targeting key recovery processes such as social connectedness, emotional regulation, and coping may further support personal recovery (Clemmensen et al., 2023; Dupont et al., 2026; Roe & Ronen, 2003). Their implementation is often limited by cost, resource constraints, and difficulties with personalization (Clemmensen et al., 2023). VR may help address these barriers by providing engaging, adaptable, and scalable recovery-oriented interventions as an adjunct to usual care (Clemmensen et al., 2023).

Inpatient settings represent a unique clinical context in which VR may encounter distinct barriers, facilitators, and outcomes (Alzahrani, 2021; Dupont et al., 2026; Roe & Ronen, 2003). Hospitalized individuals often present with acute symptom exacerbations, severe functional impairment, or elevated distress requiring intensive care (Alzahrani, 2021; Roe & Ronen, 2003). Many are also admitted involuntarily or experience coercive practices, reduced autonomy, and restricted access to stimulating environments, all of which may influence engagement in psychosocial interventions and recovery (Clemmensen et al., 2023; Dupont et al., 2026). At the organizational level, implementation is shaped by short lengths of stay, high patient turnover, staffing and space constraints, and competing clinical priorities (Dupont et al., 2026; Riches et al., 2023; Waldemar, Esbensen, Korsbek, Petersen, & Arnfred, 2018). Conversely, inpatient care also offers unique opportunities for VR implementation, including continuous clinical supervision, structured daily routines, intensive intervention schedules, and close monitoring of treatment response and safety (Raphael et al., 2021). These contextual characteristics are likely to influence not only the feasibility of VR interventions, but also the types of interventions delivered and the outcomes achieved. A clearer understanding of VR use in psychiatric inpatient settings is therefore needed to guide evidence-based implementation and identify interventions most likely to benefit hospitalized individuals.

This systematic review aimed to (1) characterize VR use in psychiatric inpatient settings, (2) document barriers and facilitators to implementation, and (3) evaluate outcomes in these settings.

## Methodology

A systematic review was conducted following PRISMA guidelines (Page et al., 2021) (PROSPERO #CRD42023446524). Relevant references were identified through searches of *Ovid*, *ScienceDirect*, *Web of Science*, *Cochrane Library, WorldCat*, *ProQuest*, and *OpenGrey.* The search covered publications from 1983 to 2025, beginning with the publication of the DSM-III and the emergence of contemporary VR systems. Keywords related to psychiatric disorders and VR were used (see Supplementary Material S1). Bibliographies of included studies, systematic reviews, and meta-analyses were also examined. Studies were included if participants were psychiatric inpatients or if at least 85% of the sample consisted of psychiatric inpatients. Detailed eligibility criteria are presented in Table 1.

**Table 1.** Inclusion and exclusion criteria Table 1. long description.

Criteria	Details
Participants	Diagnosed with a mental health disorder (DSM-III to DSM–5, ICD 9/10). Exclusion: neurodegenerative (e.g. Alzheimer’s) or neurodevelopmental (e.g. Autism spectrum) disorders.
Setting	Participants were clearly identified as psychiatric inpatients, or the sample was predominantly composed of psychiatric inpatients (≥85%). Exclusion: Studies involving mixed inpatient/outpatient samples for which inpatient-specific data could not be clearly isolated were excluded.
Intervention	Immersive 360° VR.
Publication date	1983 or later.
Study design	Any study design included. Exclusion: conference abstracts, editorials, book chapters, literature reviews (e.g. systematic reviews, meta-analyses)
Language	English, French, or Spanish

All references were imported into *Covidence* for double-blind screening. Two independent reviewers screened each article at the title/abstract and full-text stages. Discrepancies were resolved through discussion, with a third reviewer involved if needed. Corresponding authors were contacted for missing information relevant to eligibility. Methodological quality was appraised using the Mixed Methods Appraisal Tool (MMAT) (Hong et al., 2018), following the same independent review process.

Data were extracted using a pre-developed *Excel* template, pilot-tested on five articles. Extracted information (Supplementary Material S2) included study and participant characteristics, VR use details, implementation variables, and outcomes. For the first objective, VR applications were categorized based on their primary purpose: relaxation and mindfulness, social skills training, cognitive-behavioral therapy (CBT) and exposure, cognitive training, and assessment. For the second objective, implementation outcomes were classified as feasibility, acceptability, and safety. For the third objective, all reported outcomes were extracted and subsequently grouped into four domains: clinical, psychosocial, cognitive, and physiological. Clinical outcomes referred to psychiatric symptoms. Psychosocial outcomes included functioning, self-efficacy, self-esteem, readiness to change, and general well-being. Cognitive outcomes encompassed both neurocognitive (e.g. memory) and social cognitive domains (e.g. emotion recognition). Physiological outcomes included autonomic stress markers (e.g. heart rate) as well as body-related outcomes such as body mass index. Descriptive statistics and summary tables supported a narrative synthesis for all three objectives.

## Results

**Figure 1.**
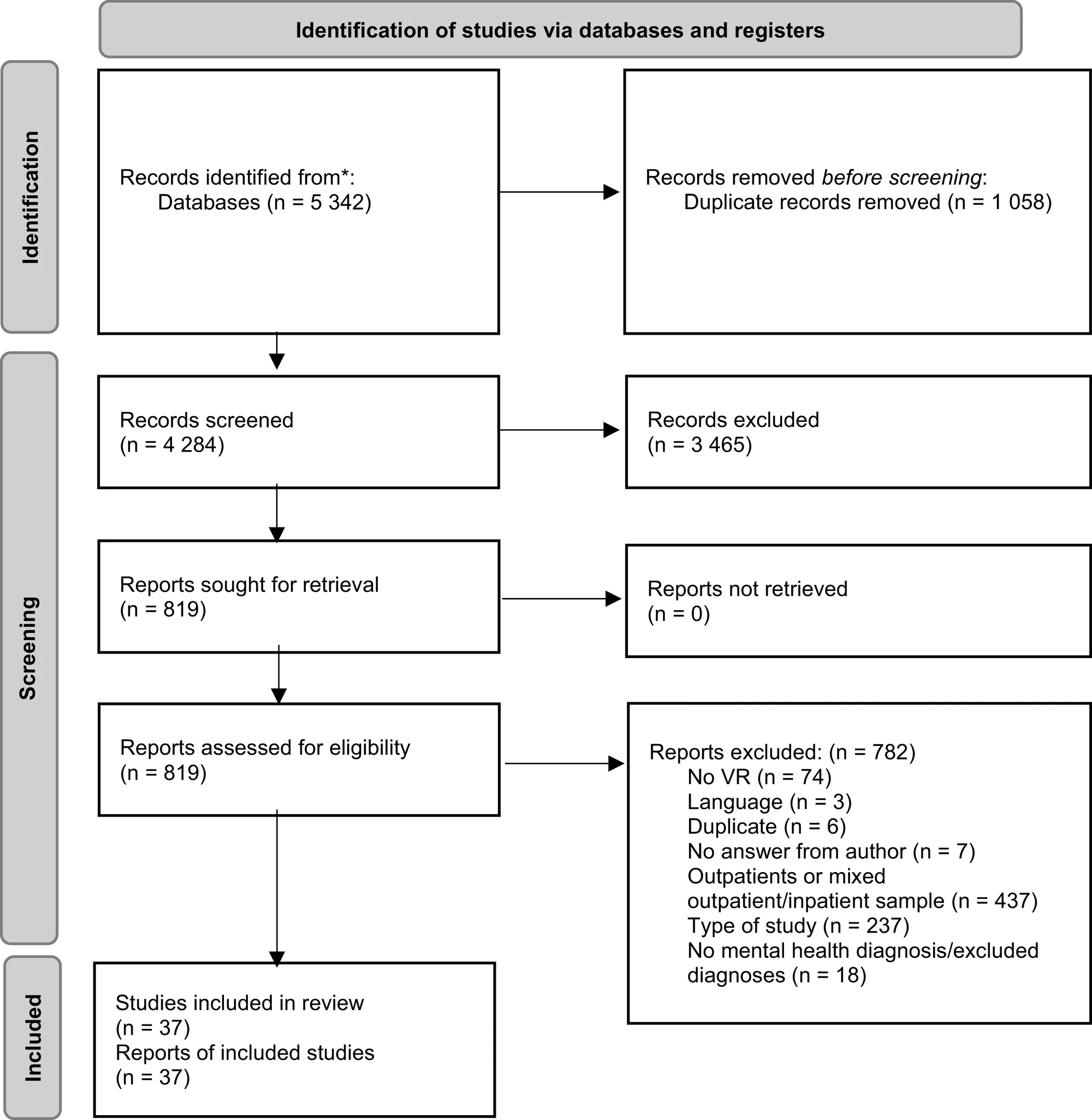
PRISMA flowchart. Figure 1. long description.

After removing duplicates, 4,284 references were identified. Title and abstract screening yielded 819 articles. Following the full-text review, 782 studies were excluded, resulting in 37 studies being included (Figure 1). Findings are presented by objective, with higher-quality evidence discussed first, followed by nonrandomized studies, pilot studies, and qualitative studies. The PRISMA checklist is provided in Supplementary Material S3, and mixed inpatient/outpatient studies excluded from the synthesis are summarized in Supplementary Material S4.

### Study characteristics

The 37 studies included 1,004 participants; characteristics are summarized in Table 2. Six diagnostic categories were identified: schizophrenia spectrum disorders (SSD [*n* = 12]), mixed psychiatric samples (*n* = 8), eating and food intake disorders (*n* = 8), substance use and addictive disorders (SUD [*n* = 6]), and mood disorders (*n* = 3). Methodological quality is summarized in Table 3, whereas VR applications and outcomes are summarized in Table 4.

**Table 2.** Demographic data Table 2. long description.

Article	*N*	Sex ratio (M:F)	Age (SD)	Diagnosis (*n*)	Duration of illness	Medication (Y/N)	Hospitalization duration (days)	Country
Borelle and Forner (2024)	7	6:1	NR	Substance-use disorder or Gambling disorder	NR	Yes	21–35	France
Bouchard et al. (2017)	Study 2: 20 Study 3: 14	Study 2: 13:7 Study 3: 7:7	Study 2: 45 (12.6) Study 3: 47 (12.8)	Gambling disorder	NR	NR	28	Canada
Brown et al. (2022)	19	12:7	NR	NR	NR	NR	NR	United Kingdom
Cesa et al. (2013)	27	0:27	32.9 (8.8)	Binge eating disorder	NR	No	35–42	Italy
Choi et al. (2010)	26	11:15	29.9 (7.9)	Schizophrenia	5.3 yrs	Yes	NR	Korea
Faulkner et al. (2024)	42	29:13	50.7 (9.6)	Alcohol use disorder	NR	Yes	34.4 (31.46)	United States
González Moraga et al. (2024)	7	6:1	36	Schizophrenia spectrum disorders and other psychotic disorders (4), Antisocial personality disorder (4), Substance use disorders (3), Personality disorder – trait specified (2), Borderline intellectual functioning (1), Autism spectrum disorder (1), Bipolar disorder, Paraphilic disorder (1)	NR	NR	NR	Sweden
Greenwald et al. (2024)	32	23:9	40	Opioid use disorder	NR	Yes	NR	United States
Holsteg et al. (2024)	8	4:3 (1 other)	35.88 (13.85)	Unipolar depression	NR	NR	NR	Germany
Ilioudi et al. (2023)	40	21:19	40.7 (14.2)	Unipolar depression (10), Anxiety disorder (10), Bipolar disorder with depressive episode (9), Bipolar disorder with hypomanic episode (10), Bipolar disorder unspecified (1)	NR	NR	NR	Sweden
Jang et al. (2005)	15	10:5	28.69 (7.06)	Schizophrenia	NR	NR	NR	Korea
Klein Tuente et al. (2020)	64	NR	39.4 (10.6)	Schizophrenia (12), Psychotic disorder not otherwise specified (6), Antisocial personality disorder (22), Borderline personality disorder (13), Narcissistic personality disorder (4), Dependent personality disorder (1), Personality disorder not otherwise specified (25), Autism spectrum disorder (5), Attention-deficit/hyperactivity disorder (11), Pedophilia (4), Other paraphilias (3)	NR	Yes	NR	Netherlands
Komemi et al. (2024)	23	19:4	33.3 (8.53)	Schizophrenia (16), Schizoaffective (7)	10.46 yrs (9.15)	Yes	28–56	Israel
Lee et al. (2009)	20	20:0	38.6 (5.9)	Alcohol use disorder	14.9 yrs (5.3)	Yes	35	Korea
Li et al. (2022)	62	39:23	39.71 (46.99)	Schizophrenia	17.44 yrs (8.94)	Yes	NR	China
Lotfinia et al. (2024)	60	60:0	37.5 (6.18)	Opioid use disorder	NR	NR	10–14	Iran
McLachlan et al. (2022)	29	9:20	15.9 (1.2)	Autism spectrum disorder (4), Adjustment disorder (1), Attention-deficit/hyperactivity disorder (2), Depression (2), Eating disorder (1); Previous psychotic episode (3), Post-traumatic stress disorder (2), Obsessive-compulsive disorder (1), No psychiatric diagnosis (14)	NR	Yes	NR	United Kingdom
Mölbert et al. (2018)	24	0:24	24 (6.35)	Anorexia nervosa	3 yrs	NR	7–112	Germany
Park et al. (2011)	33	16:17	28.1 (7.7)	Schizophrenia	6.0 yrs (5.7)	Yes	14–28	Korea
Park et al. (2009)	27	14:13	28.5 (5.7)	Schizophrenia	NR	Yes	NR	Korea
Park et al. (2009)	24	0:24	29.75 (7.25)	Paranoid schizophrenia	4.55 yrs (4.8)	Yes	7–21	Korea
Park et al. (2009)	30	16:14	28.7 (5.5)	Schizophrenia	5.8 yrs (4.3)	Yes	NR	Korea
Paslakis et al. (2017)	30	0:30	NR	Anorexia nervosa (20); Bulimia nervosa (10)	NR	No	NR	Germany
Porras-García et al. (2020)	9	NR	NR	Anorexia nervosa	NR	NR	NR	Spain
Provenzano et al. (2019)	20	0:20	23.30 (7.60)	Anorexia nervosa	NR	NR	NR	Italy
Rault et al. (2022)	13	10:3	43 (11.5)	Schizophrenia or schizoaffective disorder	NR	Yes	NR	France
Riches et al. (2023)	28	20:8	35.79 (12.55)	Mental and behavioural disorders due to psychoactive substance use (1); Schizophrenia, schizotypal, and delusional disorders (15); Mood disorders (5); Disorders of adult personality and behavior (1); Unspecified mood disorder (4); No ICD–10 diagnosis disorder recorded (1)	NR	NR	NR	United Kingdom
Riva et al. (2000)	25	0:25	NR	Binge eating disorder	NR	NR	NR	Italy
Riva et al. (2003)	36	0:36	33.07 (8.08)	Binge eating disorder	NR	No	NR	Italy
Riva et al. (2002)	20	0:20	30.5 (6.72)	Binge eating disorder	NR	No	NR	Italy
Scala et al. (2024)	20	11:9	47.85 (15.17)	Schizophrenia (*N* = 10), Bipolar disorder (*N* = 10)	NR	Yes	NR	Spain
Shah et al. (2015)	22	6:16	NR	Bipolar (12), Major depressive disorder (10)	NR	Yes	NR	Singapore
Shen et al. (2022)	26	17:9	30.81 (4.9)	Schizophrenia	NR	Yes	21–35	China
Stefaniak and Aleksandrowicz (2024)	11	NR	NR	Schizophrenia with comorbid social anxiety	NR	Yes	NR	Poland
Tan et al. (2021)	40	16:24	NR	Schizophrenia dpectrum disorder, Bipolar disorder, Major depressive disorder	NA	Yes	NR	Singapore
Van Doren et al. (2024)	20	17:3	51.55 (15.60)	Alcohol use disorder (*n* = 15), Stimulant use disorder (*n* = 7), Opioid use disorder (*n* = 2), Comorbid mental health diagnoses: Post-traumatic stress disorder (*n* = 9), Major depressive disorder (*n* = 7), Generalized anxiety disorder (*n* = 3).	NR	NR	28	United States
Wang et al. (2022)	31	14:17	24.61 (5.68)	Schizophrenia	NR	Yes	NR	China
Overall sample	1,004	446 men 473 women 1 other	35.31 years (SD = 15.82)	Schizophrenia spectrum/psychotic disorders: 329 (32.8%), Mood disorders: 65 (6.5%), Eating disorders: 180 (17.9%), Substance use disorders: 225 (22.4%), Other: 159 (15.8%)	11.45 years (7.64)	Yes = 20 (54%); No = 5 (14%); NR = 12 (32%)	28.69 days (25.26)	Italy: 17.4%; Korea: 17.1%; China: 11.9%; Other (10 countries): 53.6%

*Note*: ICD = International Statistical Classification of Diseases and Related Health Problems; NA = not applicable; NR = not reported; VR = virtual reality.

**Table 3.** Ratings of study quality Table 3. long description.

Study	Type of study	Quality of the study using the Mixed Methods Appraisal Tool				
		C1	C2	C3	C4	C5
Borelle and Forner (2024)	Qualitative study	Yes	Yes	Yes	Yes	Yes
Bouchard et al. (2017)	Randomized controlled trial	Yes	ID	Yes	ID	Yes
Brown et al. (2022)	Qualitative study	Yes	Yes	Yes	Yes	Yes
Cesa et al. (2013)	Randomized controlled trial	Yes	No	No	ID	No
Choi et al. (2010)	Cross-sectional case–control study	Yes	ID	Yes	Yes	Yes
Faulkner et al. (2024)	Randomized controlled trial	Yes	ID	No	Yes	Yes
González Moraga et al. (2024)	Qualitative study	Yes	Yes	Yes	Yes	Yes
Greenwald et al. (2024)	Nonrandomized study (single-arm)	Yes	Yes	Yes	No	Yes
Holsteg et al. (2024)	Mixed methods study	Yes	Yes	Yes	ID	Yes
Ilioudi et al. (2023)	Nonrandomized study (with control)	Yes	Yes	Yes	ID	Yes
Jang et al. (2005)	Cross-sectional observational study	ID	ID	Yes	Yes	Yes
Klein Tuente et al. (2020)	Randomized controlled trial	Yes	Yes	No	No	Yes
Komemi et al. (2024)	Nonrandomized study (single-arm)	ID	Yes	Yes	No	Yes
Lee et al. (2009)	Nonrandomized study (with control)	ID	Yes	Yes	Yes	Yes
Li et al. (2022)	Randomized controlled trial	Yes	No	Yes	Yes	Yes
Lotfinia et al. (2024)	Randomized controlled trial	Yes	Yes	ID	Yes	ID
McLachlan et al. (2022)	Nonrandomized study (single-arm)	Yes	No	Yes	No	Yes
Mölbert et al. (2018)	Cross-sectional observational study	ID	No	Yes	Yes	Yes
Park et al. (2011)	Randomized controlled trial	Yes	Yes	Yes	ID	Yes
Park, Kim, Ku, et al. (2009)	Cross-sectional observational study	No	Yes	Yes	No	Yes
Park, Ku, Park, et al. (2009)	Randomized controlled trial	Yes	Yes	No	ID	No
Park, Ku, Kim, et al. (2009)	Cross-sectional observational study	Yes	Yes	Yes	No	Yes
Paslakis et al. (2017)	Nonrandomized study (single-arm)	Yes	No	Yes	Yes	Yes
Porras-García et al. (2020)	Randomized controlled trial	ID	ID	ID	ID	ID
Provenzano et al. (2019)	Cross-sectional observational study	Yes	Yes	Yes	Yes	Yes
Rault et al. (2022)	Nonrandomized study (single-arm)	Yes	No	Yes	Yes	Yes
Riches et al. (2023)	Mixed methods study	Yes	Yes	Yes	ID	Yes
Riva et al. (2000)	Nonrandomized study (single arm)	ID	Yes	Yes	No	Yes
Riva et al. (2003)	Randomized controlled trial	Yes	Yes	Yes	Yes	Yes
Riva et al. (2002)	Randomized controlled trial	Yes	Yes	ID	Yes	Yes
Scala et al. (2024)	Cross-sectional observational study	No	Yes	Yes	Yes	ID
Shah et al. (2015)	Nonrandomized study (single-arm)	No	Yes	Yes	ID	Yes
Shen et al. (2022)	Randomized controlled trial	Yes	Yes	No	Yes	Yes
Stefaniak and Aleksandrowicz (2024)	Nonrandomized study (with control)	Yes	Yes	Yes	Yes	No
Tan et al. (2021)	Randomized controlled trial	Yes	Yes	Yes	Yes	Yes
Van Doren et al. (2024)	Mixed-methods study	Yes	Yes	Yes	ID	Yes
Wang et al. (2022)	Randomized controlled trial	Yes	Yes	Yes	Yes	Yes

**Table 4.** Summary of results Table 4. long description.

Study	VR application	Nature	Purpose	Control group	Summary of results	Effect size
Schizophrenia and related psychotic disorders						
Choi et al. (2010)	Virtual Reality System	Assessment	Assess and compare interpersonal behaviors and eye gaze patterns in emotionally positive and negative social situations.	Yes (nonclinical controls)	VR effectively assessed reduced and atypical eye gaze during VR social interactions.	NR
Jang et al. (2005)	VR Task	Assessment	Assess social anxiety response to avatars with different emotional expressions in schizophrenia	Yes (nonclinical controls)	VR avatar interactions induced social anxiety; higher negative symptoms correlated with greater anxiety for happy/neutral avatars.	NR
Komemi et al. (2024)	CR-EVR	Cognitive training	Test feasibility and initial efficacy of CR-EVR; Improve cognitive functioning and real-world functioning in schizophrenia inpatients	No	CR-EVR was feasible and well-received; Participants showed significant improvement in trained cognitive components (general cognitive functioning, functional capacity, and diversity of participation in everyday activities).	Processing speed (*r* = 0.38), Cognitive flexibility (*r* = 0.27), Verbal fluency (*r* = 0.25), Visual-motor organization (*r* = 0.25), Functional capacity: (*r* = 0.27), Diversity of participation (*r* = 0.22)
Li et al. (2022)	Virtual Reality Cognition Training System	Cognitive training	Cognitive training	Yes (TAU)	VRCTS reduced general PANSS symptoms but had no significant effect on positive or negative symptoms in participants who were in remission. Significant improvements were observed in visual learning, reasoning, and problem solving.	VRT *η*2 = 0.703
Park, Kim, Ku, et al. (2009)	Virtual reality social encounter task	Assessment	Evaluation of perception of emotion and the emotional responses in a VR social encounter	Yes (nonclinical controls)	Participants with schizophrenia underestimated the valence and arousal of angry emotions and reported higher anxiety in response to happy avatars. Unlike controls, their anxiety did not decrease from neutral to happy avatars.	NR
Park et al. (2009)	Virtual Reality Functional Skills Assessment (VRFSA)	Assessment	Receptive and expressive skills: self-introduction to a stranger, making an appointment, and how to conduct business with a co-worker	Yes (nonclinical controls)	Significant difference in VRFSA performance between participants with schizophrenia and nonclinical controls.	NR
Park et al. (2009)	Virtual reality system for measuring personal space	Assessment	Investigate characteristics of personal space in relation to the virtual person’s emotions	Yes (nonclinical controls)	People with schizophrenia maintained greater distances and wider head angles from virtual people than controls. Greater negative symptom severity was linked to increased distancing and reduced gaze toward happy and angry avatars.	NR
Park et al. (2011)	Social Skills Training with Virtual Reality	Social skills training	Conversation skills training, assertiveness skills training, and emotional expression skills training	Yes (active)	Significant improvement in social skills. Except for nonverbal skills, greater improvements observed for the VR group in conversational skills, assertiveness, interest, and generalization than controls.	NR
Rault et al. (2022)	Virtual reality relaxation therapy	Relaxation and mindfulness	Reduce anxiety and investigate the feasibility and tolerance by assessing depersonalization and cybersickness	No	Results indicate improvement in anxiety, no significant cybersickness, nor depersonalization.	NR
Shen et al. (2022)	VR-SCIT	Cognitive training	Social cognition and interaction training: emotion-perception ToM, attributional bias, and integration of learned skills	Yes (TAU and waiting list)	Participants showed improvements in social functioning, emotion recognition (especially the VR group), ToM, and metacognition, with a decrease in hostile attributional bias.	Social functioning: *d* = 1.09 Emotion recognition: *d* = 1.66 ToM: *d* = 0.42 Metacognition: *d* = 1.66 Hostile attibutional bias: *d* = 0.48
Stefaniak and Aleksandrowicz (2024)	VR Mind system	CBT and exposure therapy	Exposition to various social situation scenarios	Yes (TAU)	The VR group showed a reduction in symptom severity, social anxiety, depression, and an increase in global functioning, while levels of internalized stigma remained stable.	NR
Wang et al. (2022)	Fruit Pioneer	Cognitive training	Train on cognitive function	Yes (TAU)	Significant improvement in executive functions and memory for VR group.	Executive functions: *d* = 0.59 (task A), *d* = 0.62 (task B), Memory: *d* = 0.87
Eating and food intake disorders						
Cesa et al. (2013)	NeuroVR	CBT and exposure therapy	Enhance CBT for binge eating disorder by practicing coping and body image modification in realistic triggering situations	Yes (active and TAU)	ECT was superior to TAU and CBT in preventing weight regain and improving weight loss 1 year later; maintained low binge frequency.	NR
Mölbert et al. (2018)	NR	Assessment	Investigate body image distortion and attitudinal components in individuals with AN	Yes (non-clinical controls)	Significant differences in various measures of eating disorder and body perception between groups.	Distortion 1AFC task: *η*2 = 0.12; Distortion MoA task: *η*2 = 0.06; Sensitivity to weight loss: *η*2 = 0.06; Sensitivity to weight gain: *η*2 = 0.00; Desired change: *η*2 = 0.32; Desired-v.-actual discrepancy: *η*2 = 0.20
Paslakis et al. (2017)	Virtual Reality Jogging video	CBT and exposure therapy	Reduce acute urge to be physically active	Yes (clinical)	Significant reduction in the subjective score on the urge to move.	*d* = 0.25
Porras-García et al. (2020)	VR body exposure to eating disorder symptoms	CBT and exposure therapy	Reduce body-related attentional bias and recover a healthy weight	Yes (TAU)	Results showed a greater reduction of eating disorder symptoms, faster weight restoration, and a significant reduction in body-related attentional biases for the VR group.	Number of fixations: *η*2 = .296; BMI: *η*2 = .253
Provenzano et al. (2019)	Visuo-Tactiles Stimulation in Virtual Reality	Assessment	Characterize perceptual and cognitive-emotional components of body image distortion: body overestimation and body dissatisfaction	Yes (nonclinical controls)	Results show that individuals with anorexia nervosa have greater body dissatisfaction driven by the desire for a thinner body, but do not objectively overestimate their body size.	Effect size for the Group × Avatar interaction: *η* ^2^ = 0.119 (medium effect)
Riva et al. (2000)	Virtual Reality for Body Image Modification	CBT and exposure therapy	Treatment and assessment of obesity and binge-eating disorders	No	Significant improvements in clothing and social avoidance, overall and regional body satisfaction.	NR
Riva et al. (2002)	Virtual Reality for Eating Disorders Modification	CBT and exposure therapy	Assess and modify any stimuli that could elicit abnormal eating behavior, symptoms of anxiety related to food exposure, and the body experience of the subject	Yes (active)	VR therapy was more effective in improving participants’ overall psychological state, particularly body satisfaction, self-efficacy, and motivation for change. No significant differences were found in reducing binge eating behaviors.	NR
Riva et al. (2003)	Virtual Reality for Eating Disorders Modification	CBT and exposure therapy	Assess and modify any stimuli that could elicit abnormal eating behavior, symptoms of anxiety related to food exposure, and the body experience of the subject.	Yes (active and waiting list)	VR and CBT both reduced anxiety and depression and improved self-esteem, but VR led to greater decreases in binge eating and body image dissatisfaction.	NR
Mood disorders						
Holsteg et al. (2024)	Unity3D–based VR roleplay	Social skills training	Develop and investigate an intervention for simulation of role-play situations during supportive-expressive therapy	No	VR role-play was rated positively; barriers included technical glitches, pauses in roleplay, and burdensome system setup; the intervention was considered safe.	NR
Ilioudi et al. (2023)	Calm Place (Mimerse)	Relaxation and mindfulness	Explore effects of a VR calm room on self-reported well-being, blood pressure, and heart rate in psychiatric inpatients	Yes (active)	Both VR and physical calm rooms increased well-being and decreased heart rate, no significant differences between groups and on blood pressure.	d = 0.78
Shah et al. (2015)	VR-DE-STRESS Program	Relaxation and mindfulness	VR stress management program	No	Significant reduction in subjective stress, anxiety, skin temperature, and depression; increased perceived relaxation and knowledge about stress and stress management.	NR
Substance use disorders and addictive disorders						
Borelle and Forner (2024)	Virtual reality therapy	CBT and exposure therapy	Empower patients by stimulating craving in controlled virtual environments, allowing practice of strategies to manage addiction	No	VR therapy fosters participants’ reflexivity and control over cravings; VR is a complementary therapeutic tool, not a standalone intervention.	NR
Bouchard et al. (2017)	In virtuo	CBT and exposure therapy	Test safety and preliminary efficacy, Identify high-risk situations, Dysfunctional beliefs, Relapse prevention	Yes (Active)	Study 2: VR helped therapists identify more high-risk situations and dysfunctional thoughts associated with relapse; reduced cravings similarly to imaginal exposure; safe. Study 3: VR did not increase cravings more than control; cravings manageable; preliminary efficacy showed large symptom reductions.	Study 2: High-risk situations: *ηp*2 = 0.17 Dysfunctional thoughts: *ηp*2 = 0.08 Study 3: Measure 1: *ηp*2 = 0.46 Measure 2: *ηp*2 = 0.9 Measure 3: *ηp*2 = 0.85
Faulkner et al. (2024)	Cafeteria-like VR buffet	Assessment	Assessment of a virtual food choice experiment in a cafeteria-like VR environment	No	The VR task differentiated participants from the two treatment groups.	NR
Greenwald et al. (2024)	3D Therapy Thrive	CBT and exposure therapy	Investigate the feasibility and preliminary effect of VR, provide education to support recovery	No	VR group had lower depression, tension, and cravings at post-test, with no reported side effects.	Depressive symptoms: *d* = 1.14 Tension: *d* = 1.21 Cravings: *d* = 1.18
Lee et al. (2009)	Virtual reality alcohol treatment program	CBT and exposure therapy	Reduce craving using individualized VR alcohol cue exposure with relaxation and aversive components	Yes (Active and nonclinical controls)	VR significantly reduced alcohol craving compared to CBT/no VR and nonclinical controls, with frontal brain activity changes indicating neurobiological effects.	NR
Lotfinia et al. (2024)	Unnamed VR intervention	Relaxation and mindfulness	Investigate the effect of VR relaxation on psychological and physiological stress	Yes (Active and TAU)	Significant reduction in psychological and physiological stress when exposed to a natural VR environment.	*d* = 0.40
Mixed samples combining multiple diagnoses						
Brown et al. (2022)	gameChange	CBT and exposure	Reduce anxious avoidance of everyday social situations; prepare participants for discharge; build confidence and reduce anxiety	No	Participants and staff were enthusiastic about VR, highlighting benefits for confidence, anxiety reduction, engagement, and discharge preparation. VR was immersive and enjoyable. Concerns included suitability for acutely unwell individuals, privacy, space, equipment, and the need for training and integration into ward routines.	NR
González Moraga et al. (2024)	Virtual Reality Aggression Prevention Training (VRAPT)	Social skills training	Explore forensic psychiatric patients’ experiences of the revised VRAPT intervention through qualitative interviews	No	VR role-plays were valued for improving communication, emotion regulation, and recognition of social cues. Barriers included repetitive homework, low motivation, and technical difficulties.	NR
Klein Tuente et al. (2020)	Virtual Reality Aggression Prevention Training (VRAPT)	Social skills training	Train coping skills to reduce reactive aggression by targeting all six steps of the Social Information Processing model through immersive role-play	Yes (Waitlist)	No significant effect on primary outcomes (staff-rated and self-reported aggression) compared to TAU; short-term improvements in direct aggression, anger, impulsiveness, control, and hostility, but not maintained after 3 months.	Staff-rated aggression: *d*(pre-post) = 0.05; Self-reported aggression: *d* = 0.02; Direct aggression: *d* = 0.38; Anger: *d*(AVL) = 0.12; *d*(NAS-PI) = 0.07; Impulsiveness: *d* = 0.30; Anger control-out: *d* = 0.48; Anger control-in: *d* = 0.25; Hostility: *d* = 0.12
McLachlan et al. (2022)	InMind version 1 VR game	CBT and exposure therapy	Change individual belief regarding the ability of emotion change	No	Lower scores post-test, indicating changeable belief of emotion.	*d* = 0.78
Riches et al. (2023)	VRelax	Relaxation and mindfulness	Promote relaxation and reduce stress	No	VR improved relaxation, happiness, and connection to nature while reducing stress, anxiety, and sadness; participants and therapists responded positively; violent incidents and restrictive practices halved during implementation.	Relaxation: *d* = 1.16; Stress: *d* = −0.74; Anxiety: *d* = −0.77; Sadness: *d* = −0.54; Happiness: *d* = 1.01; Connection to nature: *d* = 1.08
Scala et al. (2024)	Dynamic virtual faces	Assessment	Measurement method of facial emotion recognition in immersive versus nonimmersive VR.	Yes (clinical and nonclinical controls)	Recognition of negative emotions was significantly impaired in both clinical groups. Both clinical groups showed similar emotion recognition accuracy in immersive and nonimmersive VR. Those with schizophrenia performed significantly worse than nonclinical controls in both settings, while those with bipolar had intermediate scores.	*d* = 1.2
Tan et al. (2021)	Virtual screen-based stress management	Relaxation and mindfulness	Psychoeducation on stress, managing stress, and relaxation techniques, practice of relaxation	Yes (Waiting list)	The VR group showed a significant increase in knowledge and perceived relaxation.	Perceived relaxation: *d* = 1.00 (session 1), *d* = 0.86 (session 2); Knowledge: *d* = 1.4 (session 1), *d* = 1.34 (session 2)
Van Doren et al. (2024)	Self-guided VR mindfulness training	Relaxation and mindfulness	Promote interoceptive awareness and reduce distractions, anxiety, and stress	No	Significant increase of positive emotion, significant decrease of negative emotion, and high levels of VR presence and state mindfulness.	Positive emotion: *d* = 1.38; Negative emotion: *d* = −1.20

*Notes*: AN = anorexia nervosa; CBT = cognitive-behavioral therapy; CUD = cannabis use disorder; DiSCoVR = Dynamic Interactive Social Cognition Training in Virtual Reality; ECT = VR-enhanced cognitive behavior therapy; MCCB = MATRICS Consensus Cognitive Battery; NR = nonreported; PANSS = Positive and Negative Syndrome Scale; SZ = schizophrenia; TAU = treatment as usual; VR = virtual reality; VR-SCIT = virtual reality-based social cognition and interaction training.

### Objective 1: Types of VR use

#### Relaxation and mindfulness

Seven studies used VR-based relaxation and mindfulness interventions. Two RCTs were identified. Lotfinia et al. (2024) compared VR environments to treatment as usual (TAU) for stress reduction in SUD. In a mixed psychiatric sample, Tan, Chng, Lau, and Klainin-Yobas (2021) assessed a VR-based stress management program.

Five nonrandomized studies extended this work by evaluating guided relaxation in SSD (Rault, Lamothe, & Pelissolo, 2022), VR compared with physical calm rooms (Ilioudi et al., 2023), and VR-based stress management (Shah et al., 2015) in mood disorders, as well as VR relaxation and mindfulness interventions in mixed psychiatric samples (Riches et al., 2023; Van Doren, Ng, Rawat, McKenna, & Blonigen, 2024).

#### Social skills training

Four studies examined VR-based social skills interventions, including two RCTs. Park et al. (2011) examined VR role-playing integrated into social skills training in SSD, and Klein Tuente et al. (2020) assessed VR aggression prevention training in forensic psychiatry.

A mixed-methods single-arm study evaluated therapist-guided VR role-plays integrated into psychodynamic psychotherapy in mood disorders (Holsteg et al., 2024), while a qualitative study explored patient experiences with VR-assisted aggression prevention training in a mixed psychiatric sample (González Moraga et al., 2024).

#### CBT and exposure therapy

Thirteen studies examined VR-based CBT and exposure interventions. Five RCTs evaluated VR as an adjunct to inpatient psychotherapy, including four in eating disorders examining VR-enhanced CBT and exposure (Cesa et al., 2013; Porras-García et al., 2020; Riva, Bacchetta, Baruffi, & Molinari, 2002; Riva, Bacchetta, Cesa, Conti, & Molinari, 2003), and one in gambling disorder integrating VR into inpatient CBT (Bouchard et al., 2017).

Six nonrandomized studies extended these findings to eating disorders, SUD, SSD with comorbid social anxiety, and mixed psychiatric samples, primarily evaluating the feasibility and preliminary effects of VR-enhanced CBT and exposure interventions (Greenwald et al., 2024; Lee et al., 2009; McLachlan, Mehdikhani, Larham, & Centifanti, 2022; Paslakis et al., 2017; Riva et al., 2000; Stefaniak & Aleksandrowicz, 2024).

Two qualitative studies examined the implementation of VR-CBT in routine psychiatric care, including one study in SUD (Borelle & Forner, 2024) and another assessing the acceptability of automated VR cognitive therapy in a mixed psychiatric sample (Brown et al., 2022).

#### Cognitive training

Four studies evaluated VR-based cognitive training in SSD, including three RCTs. Li et al. (2022) used VR cognitive remediation targeting neurocognition, while Wang, Kou, Meng, and Yu (2022) used a VR serious game to target working memory and executive functioning. Shen, Liu, Wu, Lin, and Wang (2022) used a VR version of the Social Cognition and Interaction Training (VR-SCIT) targeting social cognition and functioning.

One nonrandomized single-arm study evaluated the feasibility and preliminary efficacy of an ecological VR-based cognitive remediation program targeting neurocognition (Komemi, Tubenbaltt, Harel, Nahum, & Lipskaya-Velikovsky, 2024).

#### Assessment

Nine studies used VR for assessment. Two RCTs evaluated VR-based assessment of social competence during antipsychotic treatment in SSD (Park et al., 2009) and a VR buffet paradigm to assess food selection during pharmacological treatment for alcohol use disorder (Faulkner et al., 2024).

Seven cross-sectional studies assessed social cognition and social behavior in SSD (Choi et al., 2010; Jang et al., 2005; Park, Kim, Ku, et al., 2009), body image disturbance in eating disorders (Mölbert et al., 2018; Provenzano et al., 2019), and facial emotion recognition in immersive versus nonimmersive VR in a mixed psychiatric sample (Scala et al., 2024).

### Objective 2: Barriers and facilitators

Across study designs, VR was generally feasible, acceptable, and safe for use in psychiatric inpatient settings. However, evidence primarily originated from pilot and feasibility studies, and implementation outcomes were reported inconsistently.

#### Feasibility

Available evidence suggests that VR can be successfully implemented across psychiatric inpatient settings, with barriers mostly related to organizational factors. Common challenges included early discharge, short lengths of stay, limited availability of private spaces, competing clinical priorities, staff workload, technical difficulties, and, in some studies, COVID-19 disruptions (Brown et al., 2022; Holsteg et al., 2024; Komemi et al., 2024; Riches et al., 2023). Clinicians also emphasized the importance of staff training, technical support, and flexible scheduling (Brown et al., 2022; Holsteg et al., 2024; Riches et al., 2023). Technical specifications of VR systems are presented in Supplementary Material S5.

Despite these challenges, reported implementation indicators were encouraging. Greenwald et al. (2024) reported that 33 of 62 eligible patients (53%) enrolled in an inpatient opioid withdrawal program, and completion rates were higher in the VR group than in the TAU group (63% vs. 45%), with discontinuations primarily related to medical care or discharge rather than the intervention. Similarly, Komemi et al. (2024) reported completion of an 8-session VR cognitive remediation program by 23 of 31 participants (74%), with most attrition attributable to early discharge. Across the remaining studies, authors consistently concluded that VR could be integrated into inpatient psychiatric care when adequate organizational support and technical resources were available (Ilioudi et al., 2023; Rault et al., 2022; Stefaniak & Aleksandrowicz, 2024; Tan et al., 2021; Van Doren et al., 2024).

#### Acceptability

Acceptability was consistently high across RCTs (Ilioudi et al., 2023; Li et al., 2022), nonrandomized studies (Greenwald et al., 2024; Van Doren et al., 2024), and qualitative or mixed-methods studies (Brown et al., 2022; Holsteg et al., 2024; Riches et al., 2023). Participants described VR as engaging, immersive, enjoyable, and clinically meaningful, while facilitating relaxation, motivation, emotional engagement, and realistic yet safe opportunities to practice social interactions, emotion regulation, exposure, and everyday activities (Brown et al., 2022; Ilioudi et al., 2023; Just, Lütt, Siegle, & Döring-Brandl, 2024; McLachlan et al., 2022; Park, Kim, Ku, et al., 2009; Riches et al., 2023; Tan et al., 2021). Quantitative findings supported these observations: 94% of participants in Greenwald et al. (2024) reported satisfaction with VR, while Van Doren et al. (2024) found high ratings of satisfaction, usability, presence, confidence, and mindfulness. Similarly, Park et al. (2011) reported greater treatment interest and better generalization of social skills, and Shen et al. (2022) observed higher adherence with VR-based SCIT than with traditional SCIT.

Clinicians also viewed VR positively, highlighting its value for enhancing engagement and experiential learning (Bouchard et al., 2017; Brown et al., 2022; Holsteg et al., 2024; Riches et al., 2023; Van Doren et al., 2024). However, successful implementation depended on adequate staff training, technical support, and organizational readiness. Reported challenges included technical glitches, interruptions during role-plays, equipment maintenance, privacy considerations, and integration into routine unit activities (Brown et al., 2022; Holsteg et al., 2024; Riches et al., 2023).

#### Safety

VR was generally well tolerated across psychiatric inpatient populations, and no study reported serious adverse events directly attributable to VR. When assessed, cybersickness was uncommon and typically mild and transient, consisting primarily of headaches, dizziness, eye irritation, headset discomfort, or temporary distress (Li et al., 2022; Riches et al., 2023). Likewise, studies evaluating VR relaxation (Rault et al., 2022), CBT and exposure (Riva et al., 2000; Riva et al., 2002; Stefaniak & Aleksandrowicz, 2024), stress management (Shah et al., 2015), mindfulness (Van Doren et al., 2024), and opioid withdrawal (Greenwald et al., 2024) reported no major safety concerns.

Although several interventions intentionally elicited anxiety, craving, body-related distress, or aggressive impulses as part of therapeutic exposure, these reactions occurred within the expected therapeutic framework and were not associated with unexpected adverse events (Klein Tuente et al., 2020; Paslakis et al., 2017; Porras-García et al., 2020). Similarly, Park, Kim, Ku, et al. (2009) reported transient increases in social anxiety during VR-based social interactions in SSD, but concluded that responses reflected the emotional content of the stimuli rather than the VR technology itself. A few studies reported emotional overload or suggested that VR was unsuitable for patients who were too acutely unwell or perceived the intervention as irrelevant (Brown et al., 2022; Holsteg et al., 2024; Riches et al., 2023). Overall, reported safety concerns were primarily related to the therapeutic content and patient selection rather than to the VR technology.

### Objective 3: Treatment outcomes

#### Clinical symptoms

Higher-quality evidence from RCTs suggests mixed but generally promising effects of VR-based interventions on clinical symptoms across psychiatric diagnoses. Lower-level evidence from nonrandomized and mixed-methods studies was generally consistent with these findings.

Regarding VR-based relaxation and mindfulness, results from an RCT comparing VR-based relaxation/mindfulness with waitlist controls in a mixed psychiatric sample showed improved perceived relaxation and stress-management knowledge, with large effect sizes (Tan et al., 2021 [*N* = 40]). The results were supported by mixed-methods studies reporting large improvements in relaxation, stress, anxiety, and sadness (Riches et al., 2023 [*N* = 28]), as well as significant increases in positive affect and decreases in negative affect (Van Doren et al., 2024). In SUD, an RCT revealed that naturalistic VR environments reduced stress and cravings relative to an active control condition, although effects were small to moderate (Lotfinia et al., 2024 [*N* = 60]). Findings in mood disorders were less consistent. Ilioudi et al. (2023 [*N* = 40]) found no significant differences between VR and a physical calm room. In contrast, a single-arm study by Shah et al. (2015 [*N* = 22]) reported significant reductions in subjective stress, anxiety, and depressive symptoms, together with increased perceived relaxation and knowledge of stress management. In SSD, Rault et al. (2022 [*N* = 13]) reported improvements in anxiety in a single-arm study.

Regarding CBT/exposure therapy, four RCTs evaluated VR-enhanced CBT and exposure-based interventions in eating disorders. Overall, these studies consistently reported improvements in body-related outcomes, mood, and eating disorder psychopathology compared with TAU, conventional CBT, nutritional interventions, or waitlist controls (Porras-García et al., 2020 [*N* = 9]; Riva et al., 2002 [*N* = 20]; Riva et al., 2003 [*N* = 36]). Evidence for reductions in core eating disorder symptoms was less consistent, with one trial reporting greater reductions in binge eating following VR-based Experiential Cognitive Therapy than active control conditions (Riva et al., 2003 [*N* = 36]), whereas another found comparable improvements across treatment conditions despite greater benefits for body satisfaction and motivation for change in the VR group (Riva et al., 2002 [*N* = 20]). A preliminary RCT in anorexia nervosa further reported small improvements in urges to engage in physical activity and eating disorder symptoms following VR body exposure (Porras-García et al., 2020 [*N* = 9]). Finally, a single-arm study in binge-eating disorder reported reductions in binge eating together with improvements in body-related outcomes following VR-based experiential cognitive therapy (Riva et al., 2000 [*N* = 25]). Likewise, preliminary RCTs in gambling disorder found reductions in gambling cravings comparable with standard CBT (Bouchard et al., 2017) (Study 2, *N* = 20; Study 3, *N* = 14). In SUD, VR interventions reduced craving and negative affect, with one pilot study reporting large improvements in depressive symptoms, tension, and craving following repeated exposure to naturalistic VR environments (Greenwald et al., 2024 [*N* = 32]) and another showing greater reductions in alcohol craving than conventional CBT in alcohol use disorder (Lee et al., 2009 [*N* = 20]). In mixed psychiatric samples, McLachlan et al. (2022 [*N* = 29]) reported moderate-to-large improvements in beliefs regarding the malleability of emotions following a brief VR-based psychoeducational intervention. In SSD, VR-CBT reduced symptom severity, social anxiety, and depressive symptoms compared to TAU (Stefaniak & Aleksandrowicz, 2024 [*N* = 11]) in a nonrandomized study.

Regarding social skills training, VR-assisted aggression prevention therapy produced small-to-moderate improvements in anger control, impulsiveness, and hostility in forensic psychiatry, although effects on primary aggression outcomes were negligible and were not maintained at 3-month follow-up (Klein Tuente et al., 2020 [*N* = 64]).

#### Psychosocial outcomes

Higher-quality evidence from RCTs suggests that VR interventions may improve psychosocial functioning across several psychiatric populations.

Regarding VR-based relaxation and mindfulness, Ilioudi et al. (2023 [*N* = 40]) found no significant differences in well-being when comparing a VR calm room with a physical calm room in mood disorders, although both interventions produced moderate-to-large within-group improvements. A mixed-methods study in a mixed psychiatric sample reported large improvements in happiness, relaxation, and connectedness to nature following immersive nature-based VR (Riches et al., 2023 [*N* = 28]).

Regarding CBT, two RCTs in eating disorders showed improvements in self-efficacy, readiness to change, and self-esteem compared with TAU, conventional CBT, or waitlist controls (Riva et al., 2002 [*N* = 20]; Riva et al., 2003 [*N* = 36]). A single-arm study in binge-eating disorder also reported reduced social avoidance and greater social engagement following VR-based experiential cognitive therapy (Riva et al., 2000 [*N* = 25]). In SSD, VR-based CBT improved global functioning but did not reduce internalized stigma (Stefaniak & Aleksandrowicz, 2024 [*N* = 11]).

Regarding social skills training, one RCT in SSD showed that VR-assisted social skills training improved conversational ability and assertiveness compared with an active control group (Park et al., 2011 [*N* = 33]).

Regarding cognitive remediation, a single-arm study found that ecological VR-based cognitive remediation improved functional capacity and participation in everyday activities (Komemi et al., 2024 [*N* = 23]).

#### Cognitive outcomes

Higher-quality evidence from RCTs suggests that VR interventions improve both neurocognition and social cognition in SSD. VR-based cognitive training was associated with improvements in visual learning and reasoning/problem-solving, with a large overall treatment effect compared with TAU (Li et al., 2022 [*N* = 62]). Similarly, Wang et al. (2022 [*N* = 31]) reported moderate-to-large improvements in executive functioning and working memory following a VR serious game intervention compared with TAU. Additional evidence from an RCT supports the use of VR to target social cognition. Shen et al. (2022 [*N* = 26]) found that VR-based SCIT improved emotion recognition, theory of mind, metacognition, and social functioning while reducing hostile attribution bias compared with traditional SCIT and a waiting-list control.

Consistent preliminary evidence was provided in a single-arm study by Komemi et al. (2024 [*n* = 23]) who reported small-to-moderate improvements across several neurocognitive domains, including processing speed, cognitive flexibility, verbal fluency, and functional capacity following ecological VR-based cognitive remediation.

#### Physiological outcomes

Higher-quality evidence from RCTs suggests that VR-based interventions may improve selected physiological outcomes, although findings remain limited.

Regarding VR-based relaxation and mindfulness, naturalistic VR environments improved physiological indicators of stress, including respiratory rate and skin conductance in an RCT with SUD compared with active and passive control conditions (Lotfinia et al., 2024 [*N* = 60]). In a mixed psychiatric sample, Tan et al. (2021 [*N* = 40]) found no clear effects on objective physiological stress markers compared to a waiting-list control in an RCT despite improvements in perceived relaxation and stress-management knowledge. In mood disorders, Shah et al. (2015 [*N* = 22]) reported improvements in physiological markers of relaxation in a single-arm study, including increased skin temperature, following a VR-based stress management program. Similarly, in a nonrandomized study comparing a VR calm room with a physical calm room, Ilioudi et al. (2023 [*N* = 40]) found no significant between-group differences for physiological indicators such as heart rate or blood pressure.

Regarding CBT, a small number of studies examined body-related outcomes in eating disorders. In binge-eating disorder, an RCT found that VR-enhanced CBT was associated with better maintenance of weight loss and lower relapse rates at 1-year follow-up compared with TAU (Cesa et al., 2013 [*N* = 27]). Similarly, a preliminary RCT in anorexia nervosa reported improvements in BMI and body-related attentional bias following VR body exposure compared with non-VR interventions, with small-to-moderate effects (Porras-García et al., 2020 [*N* = 9]).

## Discussion

This systematic review synthesized evidence regarding the use, feasibility, acceptability, safety, and effects of VR in psychiatric inpatient settings. Overall, findings indicate that VR has been applied across diverse psychiatric diagnoses and therapeutic contexts, including relaxation/mindfulness, social skills training, CBT/exposure, cognitive training, and assessment. These applications largely mirror those reported in outpatient settings (Freeman et al., 2017; Maples-Keller et al., 2017; Valmaggia et al., 2016). However, CBT/exposure and assessment accounted for most studies, reflecting the current stage of VR implementation in psychiatry (Benbow & Anderson, 2019; Carl et al., 2019; Fernández-Álvarez et al., 2019).

Across studies, VR was associated with improvements in clinical, psychosocial, cognitive, and physiological outcomes and was generally feasible, acceptable, and safe for both inpatients and clinicians. Nevertheless, these findings should be interpreted cautiously given the substantial heterogeneity in VR modalities, psychiatric populations, intervention protocols, and methodological quality.

### Benefits of VR-based treatments in inpatient settings

Overall, VR-based interventions demonstrated benefits across multiple outcome domains despite the greater clinical acuity typically encountered in inpatient settings. Clinical improvements were reported across SSD, mood disorders, eating disorders, SUD, and mixed psychiatric samples, while psychosocial benefits included enhanced interpersonal functioning, well-being, relaxation, and self-efficacy. These findings are consistent with previous outpatient-focused reviews (Carl et al., 2019; *G. Riva, Wiederhold, & Mantovani, 2019*; Wechsler, Kümpers, & Mühlberger, 2019) and the recent meta-analysis by Zeka et al. (2025), which concluded that VR interventions may benefit several psychiatric disorders while emphasizing that the certainty of the evidence remains low to moderate because of methodological limitations. The immersive and interactive characteristics of VR may partly explain these findings by promoting presence and facilitating safe engagement with avoided or emotionally salient stimuli (Riva et al., 2019). Such features may be particularly valuable in inpatient care, where emotional dysregulation, reduced motivation, and avoidance can limit participation in conventional psychosocial interventions (Clemmensen et al., 2023; Roe & Ronen, 2003).

VR also showed promise for cognitive remediation in inpatient care. Consistent with the previous meta-analysis of inpatient cognitive remediation by Cella et al. (2020), RCTs demonstrated improvements in neurocognition and social cognition (Li et al., 2022; Shen et al., 2022; Wang et al., 2022). Beyond improving cognitive functioning, VR may provide a more engaging and ecologically valid platform for practicing cognitive and social cognitive skills than conventional approaches.

Evidence for physiological effects was more limited, but suggests that VR may influence autonomic stress response alongside psychological outcomes (Lotfinia et al., 2024; Porras-García et al., 2020; Shah et al., 2015). However, the durability of these effects remains uncertain because most studies assessed only short-term outcomes and some benefits diminished at follow-up. Future studies should therefore examine post-discharge maintenance and long-term effects on functioning, symptoms, and quality of life. Although beneficial effects were observed across several diagnoses, evidence remains preliminary.

### Feasibility, acceptability, and safety

Overall, VR interventions were found to be feasible, acceptable, and safe in psychiatric inpatient settings. Users reported high levels of satisfaction, realism, and engagement, and most completed the interventions. Reported dropout rates were low, which may have been supported by the gamification component of VR but also by the inpatient setting where structured routines and real-time monitoring facilitate adherence and individualized delivery (Balachander et al., 2020; Raphael et al., 2021). Further, successful implementation depended primarily on organizational factors rather than on the technology itself. Consistent with previous reviews (e.g. Maples-Keller et al., 2017), adequate staff training, technical support, and institutional readiness emerged as key facilitators, whereas limited staffing, technical difficulties, equipment maintenance, and restricted physical space represented the principal barriers (Raphael et al., 2021; Riva et al., 2019). These findings suggest that implementation efforts should focus as much on organizational readiness as on the technology itself.

Although occasional emotional overload or transient symptom exacerbation was reported, these events should be interpreted within the therapeutic context of the interventions. Several VR applications intentionally incorporated exposure to anxiety-provoking, emotionally salient, or craving-related stimuli as part of evidence-based CBT/exposure. Consequently, temporary increases in distress or emotional activation likely reflect the expected therapeutic process rather than adverse effects of VR technology. The distinction may be particularly relevant in psychiatric inpatient settings, where patients often present with greater clinical instability than outpatient populations. Appropriate patient selection, clinician supervision, gradual exposure, and structured debriefing therefore remain essential to maximize therapeutic benefits while minimizing unnecessary distress (Maples-Keller et al., 2017; Riches et al., 2023).

### Implications for implementation of VR in inpatient care

This review highlights several practical considerations for implementing VR in psychiatric inpatient care. First, clinician training and access to technical support appear essential for successful adoption. Second, user-friendly and clinically accessible VR applications may facilitate implementation by reducing technical complexity and staff burden. Third, staffing constraints remain an important challenge, although certain interventions, such as relaxation/mindfulness, and cognitive training may be delivered by nonspecialized staff following appropriate training, potentially improving feasibility and accessibility. Finally, future studies should clarify the influence of psychoactive medications and symptom severity on the safety and effectiveness of VR-based interventions. Although no evidence of serious harm was identified, these factors remain largely unexplored.

### Review strengths and limitations

This review followed PRISMA guidelines and included a comprehensive search of peer-reviewed and gray literature, reducing the risk of publication bias. Study quality was generally adequate according to the MMAT. Another strength is the geographical diversity of the included studies. Although VR may be perceived as a technology used in high-income countries, several studies originated from diverse settings, including Brazil, China, and Iran, suggesting growing global interest in VR applications for psychiatric care.

Several limitations should also be acknowledged. Inconsistent reporting of inpatient status and the absence of author responses resulted in the exclusion of potentially relevant studies. Excluding study protocols may also have omitted forthcoming evidence. Additionally, many studies included small and heterogeneous samples, and the diversity of interventions, outcomes, study designs, and psychiatric diagnoses precluded quantitative synthesis and limited direct comparisons across studies. Unlike broader meta-analyses (e.g. Zeka et al., 2025), this review specifically focused on psychiatric inpatient settings and therefore adopted a narrative synthesis to capture the diversity of interventions, implementation factors, and study designs. Publication bias also cannot be excluded, as studies with positive findings may have been more likely to be published. Finally, limited reporting of hospitalization duration prevented examination of how length of stay may influence VR implementation, dosage, or outcomes.

## Conclusion

This review found that VR has been applied across a broad range of psychiatric inpatient settings and that available evidence generally supports its feasibility, acceptability, and safety while suggesting potential benefits across clinical, psychosocial, cognitive, and physiological outcomes. However, the current evidence remains preliminary because of small sample sizes, methodological heterogeneity, and limited long-term follow-up. VR therefore appears to represent a promising complement to, rather than a replacement for, conventional inpatient care. Future research should prioritize adequately powered trials, standardized intervention and safety procedures, and long-term follow-up to better define the role of VR in psychiatric inpatient practice.

## Supporting information

10.1017/S0033291726105662.sm001 Fontaine-Dussault et al. supplementary material Fontaine-Dussault et al. supplementary material

## Supplementary material

The supplementary material for this article can be found at http://doi.org/10.1017/S0033291726105662.

## Long descriptions

### Table 1. Long description

The table consists of two columns: Criteria and Details.

* Participants: Includes those diagnosed with a mental health disorder (D S M-III to D S M-5, I C D 9/10). Exclusion: neurodegenerative (e.g. Alzheimer’s) or neurodevelopmental (e.g. Autism spectrum) disorders.

* Setting: Participants clearly identified as psychiatric inpatients, or the sample was predominantly composed of psychiatric inpatients (greater than or equal to 85 percent). Exclusion: Studies involving mixed inpatient/outpatient samples where inpatient-specific data could not be isolated.

* Intervention: Immersive 360 degree V R.

* Publication date: 1983 or later.

* Study design: Any study design included. Exclusion: conference abstracts, editorials, book chapters, literature reviews (e.g. systematic reviews, meta-analyses).

* Language: English, French, or Spanish.

Navigate back to Table 1..

### Figure 1. Long description

The flowchart is organized into three vertical phases labeled on the left: Identification, Screening, and Included.

1. Identification Phase:

* Top box: Identification of studies via databases and registers.

* Left box: Records identified from Databases (n = 5 342).

* Right box (exclusion): Records removed before screening: Duplicate records removed (n = 1 058).

2. Screening Phase:

* Left box: Records screened (n = 4 284).

* Right box (exclusion): Records excluded (n = 3 465).

* Left box: Reports sought for retrieval (n = 819).

* Right box (exclusion): Reports not retrieved (n = 0).

* Left box: Reports assessed for eligibility (n = 819).

* Right box (exclusion): Reports excluded (n = 782). Reasons include: No V R (n = 74), Language (n = 3), Duplicate (n = 6), No answer from author (n = 7), Outpatients or mixed outpatient/inpatient sample (n = 437), Type of study (n = 237), and No mental health diagnosis/excluded diagnoses (n = 18).

3. Included Phase:

* Final box: Studies included in review (n = 37) and Reports of included studies (n = 37).

Navigate back to Figure 1..

### Table 2. Long description

The table contains 9 columns: Article, N, Sex ratio (M:F), Age (S D), Diagnosis (n), Duration of illness, Medication (Y/N), Hospitalization duration (days), and Country.

Key entries include:

* Borelle and Forner (2024): 7 participants, 6:1 sex ratio, Substance-use or Gambling disorder, France.

* Bouchard et al. (2017): 34 participants across two studies, Gambling disorder, Canada.

* Cesa et al. (2013): 27 females, Binge eating disorder, Italy.

* Choi et al. (2010): 26 participants, Schizophrenia, Korea.

* Faulkner et al. (2024): 42 participants, Alcohol use disorder, United States.

* Klein Tuente et al. (2020): 64 participants, multiple diagnoses including Schizophrenia and Personality disorders, Netherlands.

* Li et al. (2022): 62 participants, Schizophrenia, China.

* Lotfinia et al. (2024): 60 males, Opioid use disorder, Iran.

* Overall sample: 1,004 total participants (446 men, 473 women, 1 other). Mean age is 35.31 years (S D = 15.82). Diagnoses distribution: Schizophrenia spectrum/psychotic disorders 32.8 percent, Mood disorders 6.5 percent, Eating disorders 17.9 percent, Substance use disorders 22.4 percent, and Other 15.8 percent. Geographic distribution: Italy 17.4 percent, Korea 17.1 percent, China 11.9 percent, and Other countries 53.6 percent.

Abbreviations used: I C D for International Statistical Classification of Diseases and Related Health Problems, N A for not applicable, N R for not reported, and V R for virtual reality.

Navigate back to Table 2..

### Table 3. Long description

The table lists 37 studies with their respective study types and quality ratings (Yes, No, or I D) for five criteria labeled C 1 through C 5.

* Borelle and Forner (2024), Qualitative study: Yes for all criteria.

* Bouchard et al. (2017), Randomized controlled trial: Yes for C 1, C 3, C 5; I D for C 2, C 4.

* Brown et al. (2022), Qualitative study: Yes for all criteria.

* Cesa et al. (2013), Randomized controlled trial: Yes for C 1; No for C 2, C 3, C 5; I D for C 4.

* Choi et al. (2010), Cross-sectional case-control study: Yes for C 1, C 3, C 4, C 5; I D for C 2.

* Faulkner et al. (2024), Randomized controlled trial: Yes for C 1, C 4, C 5; I D for C 2; No for C 3.

* González Moraga et al. (2024), Qualitative study: Yes for all criteria.

* Greenwald et al. (2024), Nonrandomized study (single-arm): Yes for C 1, C 2, C 3, C 5; No for C 4.

* Holsteg et al. (2024), Mixed methods study: Yes for C 1, C 2, C 3, C 5; I D for C 4.

* Ilioudi et al. (2023), Nonrandomized study (with control): Yes for C 1, C 2, C 3, C 5; I D for C 4.

* Jang et al. (2005), Cross-sectional observational study: I D for C 1, C 2; Yes for C 3, C 4, C 5.

* Klein Tuente et al. (2020), Randomized controlled trial: Yes for C 1, C 2, C 5; No for C 3, C 4.

* Komemi et al. (2024), Nonrandomized study (single-arm): I D for C 1; Yes for C 2, C 3, C 5; No for C 4.

* Lee et al. (2009), Nonrandomized study (with control): I D for C 1; Yes for C 2, C 3, C 4, C 5.

* Li et al. (2022), Randomized controlled trial: Yes for C 1, C 3, C 4, C 5; No for C 2.

* Lotfinia et al. (2024), Randomized controlled trial: Yes for C 1, C 2, C 4; I D for C 3, C 5.

* McLachlan et al. (2022), Nonrandomized study (single-arm): Yes for C 1, C 3, C 5; No for C 2, C 4.

* Mölbert et al. (2018), Cross-sectional observational study: I D for C 1; No for C 2; Yes for C 3, C 4, C 5.

* Park et al. (2011), Randomized controlled trial: Yes for C 1, C 2, C 3, C 5; I D for C 4.

* Park, Kim, Ku, et al. (2009), Cross-sectional observational study: No for C 1, C 4; Yes for C 2, C 3, C 5.

* Park, Ku, Park, et al. (2009), Randomized controlled trial: Yes for C 1, C 2; No for C 3, C 5; I D for C 4.

* Park, Ku, Kim, et al. (2009), Cross-sectional observational study: Yes for C 1, C 2, C 3, C 5; No for C 4.

* Paslakis et al. (2017), Nonrandomized study (single-arm): Yes for C 1, C 3, C 4, C 5; No for C 2.

* Porras-García et al. (2020), Randomized controlled trial: I D for all criteria.

* Provenzano et al. (2019), Cross-sectional observational study: Yes for all criteria.

* Rault et al. (2022), Nonrandomized study (single-arm): Yes for C 1, C 3, C 4, C 5; No for C 2.

* Riches et al. (2023), Mixed methods study: Yes for C 1, C 2, C 3, C 5; I D for C 4.

* Riva et al. (2000), Nonrandomized study (single arm): I D for C 1; Yes for C 2, C 3, C 5; No for C 4.

* Riva et al. (2003), Randomized controlled trial: Yes for all criteria.

* Riva et al. (2002), Randomized controlled trial: Yes for C 1, C 2, C 4, C 5; I D for C 3.

* Scala et al. (2024), Cross-sectional observational study: No for C 1; Yes for C 2, C 3, C 4; I D for C 5.

* Shah et al. (2015), Nonrandomized study (single-arm): No for C 1; Yes for C 2, C 3, C 5; I D for C 4.

* Shen et al. (2022), Randomized controlled trial: Yes for C 1, C 2, C 4, C 5; No for C 3.

* Stefaniak and Aleksandrowicz (2024), Nonrandomized study (with control): Yes for C 1, C 2, C 3, C 4; No for C 5.

* Tan et al. (2021), Randomized controlled trial: Yes for all criteria.

* Van Doren et al. (2024), Mixed-methods study: Yes for C 1, C 2, C 3, C 5; I D for C 4.

* Wang et al. (2022), Randomized controlled trial: Yes for all criteria.

Navigate back to Table 3..

### Table 4. Long description

The table is organized into five main diagnostic categories.

1. Schizophrenia and related psychotic disorders: Includes 12 studies (Choi et al. 2010 to Wang et al. 2022). Applications include assessment, cognitive training, and social skills training. Key results show V R is effective for assessing atypical eye gaze, inducing social anxiety for assessment, and improving cognitive functioning and social skills. Effect sizes range from r = 0.22 to eta-squared = 0.703.

2. Eating and food intake disorders: Includes 8 studies (Cesa et al. 2013 to Riva et al. 2003). Applications focus on C B T and exposure therapy for binge eating, anorexia nervosa, and obesity. Results indicate V R helps prevent weight regain and reduces body-related attentional bias. Effect sizes include d = 0.25 and eta-squared values up to 0.32.

3. Mood disorders: Includes 3 studies (Holsteg et al. 2024, Ilioudi et al. 2023, Shah et al. 2015). Applications cover roleplay, relaxation, and mindfulness. Results show increased well-being and reduced stress/anxiety, with one study reporting d = 0.78.

4. Substance use disorders and addictive disorders: Includes 6 studies (Borelle and Forner 2024 to Lotfinia et al. 2024). Applications use C B T and exposure to manage cravings. Results show significant reductions in alcohol and substance cravings. Effect sizes include d = 0.40 and eta-p-squared up to 0.9.

5. Mixed samples combining multiple diagnoses: Includes 8 studies (Brown et al. 2022 to Van Doren et al. 2024). Applications include aggression prevention training (V R A P T), relaxation, and emotion recognition assessment. Results show improved communication, reduced violent incidents in wards, and increased state mindfulness. Effect sizes range from d = 0.02 to d = 1.4.

Navigate back to Table 4..
