# Supplementary material for: Applications and outcomes of virtual reality in inpatient psychiatry: A systematic review
Source: Psychol Med. 2026 Sep 3;56:e260. doi: 10.1017/S0033291726105662 (PMC13735849; doi:10.1017/S0033291726105662)
Supplement: Supplementary file 1 [file S0033291726105662sup001.docx]

**Supplementary materials**

**Supplement 1: Search Keywords**

Keywords related to psychiatric disorders and VR were used. No keywords targeted the patient’s status (inpatient) since this information was not always provided directly in the article and needed to be infer from the paper or the authors were contacted to obtain this information.

**Example of strategy search in Pubmed:**

("Virtual Reality" OR "Virtual Reality Therapy" OR "Virtual Reality Therapies" OR "Virtual realities" OR "Virtual Reality Exposure Therapy" OR "Virtual Reality Immersion Therapy" OR "Virtual Reality Exercise" OR "Virtual Reality Exercises")

AND

("Psychiatric Disorder" OR "Psychiatric Disorders" OR "Behavior Disorders" OR "Psychiatric Diagnosis" OR "Severe Mental Disorder" OR "Severe Mental Disorders" OR "Mental Disorder" OR "Psychiatric Illness" OR "Psychiatric Illnesses" OR "Psychiatric Disease" OR "Psychiatric Diseases" OR "Mental Illness" OR "Mental Illnesses" OR schizophrenia OR "schizoaffective disorder" OR "Schizophrenic Disorder" OR "Schizoaffective Disorders" OR "Psychotic Disorder" OR Psychosis OR Psychoses OR "Schizophreniform Disorder" OR "first-episode psychosis" OR "Schizophrenia and Disorders with Psychotic Features" OR "Bipolar disorder" OR "Manic-Depressive Psychosis" OR "Manic Depressive Psychosis" OR "Affective psychosis" OR "Bipolar Affective Psychosis" OR "Bipolar Disorder Type 1" OR "Bipolar Disorder Type 2" OR "Manic Depression" OR "Bipolar Depression" OR "Type 1 bipolar disorder" OR "Type 2 bipolar disorder" OR "Manic disorder" OR "Anxiety disorder" OR "Neurotic Anxiety State" OR "Depressive disorder" OR "Unipolar Depression" OR "Anorexia Nervosa" OR "Avoidant Restrictive Food Intake Disorder" OR "Binge-Eating Disorder" OR "Bulimia Nervosa" OR "Personality Disorder" OR "Avoidant Personality Disorder" OR "Narcissistic Personality Disorder" OR "Antisocial Personality Disorder" OR "Borderline Personality Disorder" OR "Compulsive Personality Disorder" OR "Dependent Personality Disorder" OR "Histrionic Personality Disorder" OR "Paranoid Personality Disorder" OR "Schizoid Personality Disorder" OR "Schizotypal Personality Disorder" OR "Obsessive compulsive disorder" OR "Post-Traumatic Stress Disorder" OR PTSD OR "Post Traumatic Stress Disorder" OR "Posttraumatic Stress Disorder" OR "Acute Post-Traumatic Stress Disorder" OR "Substance Related Disorder" OR "Drug Use Disorder" OR "Substance Abuse" OR "Substance Dependence" OR "Substance Addiction" OR "Drug Dependence" OR "Drug Addiction" OR "Substance Use" OR "Drug Abuse" OR "Substance Use Disorder")

**Supplement 2:** **Data Extraction Items**

The following information was extracted from each article

1) Article characteristics
   a. Title
   b. Year
   c. Authors
2) Participant characteristics
   a. Sample size
   b. Age (mean, SD)
   c. Sex ratio (n)
   d. Ethnicity (n)
   e. Diagnosis (n)
   f. Duration of illness (mean, SD)
   g. Age at onset of illness (mean, SD)
   h. Number of hospitalizations (mean, SD)
   i. Medication (Y/N)
   j. Chlorpromazine equivalent (mean, SD)
3) VR intervention details
   a. Application name
   b. Nature of the intervention (e.g., CBT and exposure, Assessment)
   c. Purpose
   d. Type of headset
   e. Control group (Y/N; TAU vs. Active)
4) Implementation variables
   a. Feasibility (e.g., number of sessions completed, number of missed sessions, duration of session, completion of intervention)
   b. Acceptability (e.g., participants’ appreciation, health care professionals’ opinion)
   c. Safety (e.g., cybersickness, injuries, adverse and unwanted events)
5) VR-related outcomes
   a. Clinical
   b. Psychosocial
   c. Cognitive
   d. Physiological

**Supplement 3: PRISMA Checklist - Abstract**

| **Section and Topic** | **Item #** | **Checklist item** | **Reported (Yes/No)** |
| --- | --- | --- | --- |
| **TITLE** | | |  |
| Title | 1 | Identify the report as a systematic review. | Yes |
| **BACKGROUND** | | |  |
| Objectives | 2 | Provide an explicit statement of the main objective(s) or question(s) the review addresses. | Yes |
| **METHODS** | | |  |
| Eligibility criteria | 3 | Specify the inclusion and exclusion criteria for the review. | Yes |
| Information sources | 4 | Specify the information sources (e.g. databases, registers) used to identify studies and the date when each was last searched. | Yes |
| Risk of bias | 5 | Specify the methods used to assess risk of bias in the included studies. | Yes |
| Synthesis of results | 6 | Specify the methods used to present and synthesise results. | Yes |
| **RESULTS** | | |  |
| Included studies | 7 | Give the total number of included studies and participants and summarise relevant characteristics of studies. | Yes |
| Synthesis of results | 8 | Present results for main outcomes, preferably indicating the number of included studies and participants for each. If meta-analysis was done, report the summary estimate and confidence/credible interval. If comparing groups, indicate the direction of the effect (i.e. which group is favoured). | Yes |
| **DISCUSSION** | | |  |
| Limitations of evidence | 9 | Provide a brief summary of the limitations of the evidence included in the review (e.g. study risk of bias, inconsistency and imprecision). | Yes |
| Interpretation | 10 | Provide a general interpretation of the results and important implications. | Yes |
| **OTHER** | | |  |
| Funding | 11 | Specify the primary source of funding for the review. | N/A |
| Registration | 12 | Provide the register name and registration number. | Yes |

**Supplement 3: PRISMA Checklist**

| **Section and Topic** | **Item #** | **Checklist item** | **Location where item is reported** |
| --- | --- | --- | --- |
| **TITLE** | | |  |
| Title | 1 | Identify the report as a systematic review. | Page 1 |
| **ABSTRACT** | | |  |
| Abstract | 2 | See the PRISMA 2020 for Abstracts checklist. | Page 2 |
| **INTRODUCTION** | | |  |
| Rationale | 3 | Describe the rationale for the review in the context of existing knowledge. | Pages 3-4 |
| Objectives | 4 | Provide an explicit statement of the objective(s) or question(s) the review addresses. | Page 4 |
| **METHODS** | | |  |
| Eligibility criteria | 5 | Specify the inclusion and exclusion criteria for the review and how studies were grouped for the syntheses. | Table 1, Page 5 |
| Information sources | 6 | Specify all databases, registers, websites, organisations, reference lists and other sources searched or consulted to identify studies. Specify the date when each source was last searched or consulted. | Page 4 |
| Search strategy | 7 | Present the full search strategies for all databases, registers and websites, including any filters and limits used. | Page 4, Supplement 1 |
| Selection process | 8 | Specify the methods used to decide whether a study met the inclusion criteria of the review, including how many reviewers screened each record and each report retrieved, whether they worked independently, and if applicable, details of automation tools used in the process. | Page 5 |
| Data collection process | 9 | Specify the methods used to collect data from reports, including how many reviewers collected data from each report, whether they worked independently, any processes for obtaining or confirming data from study investigators, and if applicable, details of automation tools used in the process. | Page 5 |
| Data items | 10a | List and define all outcomes for which data were sought. Specify whether all results that were compatible with each outcome domain in each study were sought (e.g. for all measures, time points, analyses), and if not, the methods used to decide which results to collect. | Page 5, Supplement 2 |
|  | 10b | List and define all other variables for which data were sought (e.g. participant and intervention characteristics, funding sources). Describe any assumptions made about any missing or unclear information. | Page 5, Supplement 2 |
| Study risk of bias assessment | 11 | Specify the methods used to assess risk of bias in the included studies, including details of the tool(s) used, how many reviewers assessed each study and whether they worked independently, and if applicable, details of automation tools used in the process. | Page 5 |
| Effect measures | 12 | Specify for each outcome the effect measure(s) (e.g. risk ratio, mean difference) used in the synthesis or presentation of results. | Page 5 |
| Synthesis methods | 13a | Describe the processes used to decide which studies were eligible for each synthesis (e.g. tabulating the study intervention characteristics and comparing against the planned groups for each synthesis (item #5)). | Page 5 |
|  | 13b | Describe any methods required to prepare the data for presentation or synthesis, such as handling of missing summary statistics, or data conversions. | Page 5 |
|  | 13c | Describe any methods used to tabulate or visually display results of individual studies and syntheses. | Page 5 |
|  | 13d | Describe any methods used to synthesize results and provide a rationale for the choice(s). If meta-analysis was performed, describe the model(s), method(s) to identify the presence and extent of statistical heterogeneity, and software package(s) used. | Page 5 |
|  | 13e | Describe any methods used to explore possible causes of heterogeneity among study results (e.g. subgroup analysis, meta-regression). | N/A |
|  | 13f | Describe any sensitivity analyses conducted to assess robustness of the synthesized results. | N/A |
| Reporting bias assessment | 14 | Describe any methods used to assess risk of bias due to missing results in a synthesis (arising from reporting biases). | Pages 5 |
| Certainty assessment | 15 | Describe any methods used to assess certainty (or confidence) in the body of evidence for an outcome. | Pages 5 |
| **RESULTS** | | |  |
| Study selection | 16a | Describe the results of the search and selection process, from the number of records identified in the search to the number of studies included in the review, ideally using a flow diagram. | Page 6, Figure 1 |
|  | 16b | Cite studies that might appear to meet the inclusion criteria, but which were excluded, and explain why they were excluded. | Page 6, Supplement 4 |
| Study characteristics | 17 | Cite each included study and present its characteristics. | Tables 2, 3 and 4 |
| Risk of bias in studies | 18 | Present assessments of risk of bias for each included study. | Table 3 |
| Results of individual studies | 19 | For all outcomes, present, for each study: (a) summary statistics for each group (where appropriate) and (b) an effect estimate and its precision (e.g. confidence/credible interval), ideally using structured tables or plots. | Table 4 |
| Results of syntheses | 20a | For each synthesis, briefly summarise the characteristics and risk of bias among contributing studies. | Table 3 |
|  | 20b | Present results of all statistical syntheses conducted. If meta-analysis was done, present for each the summary estimate and its precision (e.g. confidence/credible interval) and measures of statistical heterogeneity. If comparing groups, describe the direction of the effect. | Pages 6-13, Tables 2 and 4 |
|  | 20c | Present results of all investigations of possible causes of heterogeneity among study results. | Table 3 |
|  | 20d | Present results of all sensitivity analyses conducted to assess the robustness of the synthesized results. | N/A |
| Reporting biases | 21 | Present assessments of risk of bias due to missing results (arising from reporting biases) for each synthesis assessed. | Table 3 |
| Certainty of evidence | 22 | Present assessments of certainty (or confidence) in the body of evidence for each outcome assessed. | Table 3 |
| **DISCUSSION** | | |  |
| Discussion | 23a | Provide a general interpretation of the results in the context of other evidence. | Pages 15-19 |
|  | 23b | Discuss any limitations of the evidence included in the review. | Page 19 |
|  | 23c | Discuss any limitations of the review processes used. | Page 19 |
|  | 23d | Discuss implications of the results for practice, policy, and future research. | Page 19 |
| **OTHER INFORMATION** | | |  |
| Registration and protocol | 24a | Provide registration information for the review, including register name and registration number, or state that the review was not registered. | Pages 2 and 4 |
|  | 24b | Indicate where the review protocol can be accessed, or state that a protocol was not prepared. | Pages 2 and 4 |
|  | 24c | Describe and explain any amendments to information provided at registration or in the protocol. | Page 5 |
| Support | 25 | Describe sources of financial or non-financial support for the review, and the role of the funders or sponsors in the review. | N/A |
| Competing interests | 26 | Declare any competing interests of review authors. | N/A |
| Availability of data, code and other materials | 27 | Report which of the following are publicly available and where they can be found: template data collection forms; data extracted from included studies; data used for all analyses; analytic code; any other materials used in the review. | N/A |

**Supplement 4: Mixed inpatient/outpatient studies excluded from the synthes**

**Table 1. Demographic data of mixed in- and outpatient studies**

| **Article** | **N** | **Sample status** | **Sex ratio (M:F)** | **Age (SD)** | **Diagnosis (n)** | **Duration of illness** | **Medication (Y/N)** | **Hospit. duration** | **Country** |
| --- | --- | --- | --- | --- | --- | --- | --- | --- | --- |
| Altunkaya et al. (2022) | 346 | 1.2% inpatients; 98.8% outpatients | 232:114 | 37.2 (12.5) | Schizophrenia spectrum disorder or Affective diagnosis with psychotic symptoms | NR | Yes | NR | United Kingdom |
| Da Costa & de Carvalho (2004) | 4 | 0% inpatients 100% outpatients | 03:01 | 45.25 (8.66) | Schizophrenia | NR | Yes | NR | Brazil |
| Dietrichkeit et al. (2020) | 39 | 51.3% inpatients; 48.7% outpatients | 24:15 | 34.72 (8.68) | Schizophrenia | NR | Yes | NR | Germany |
| Fisher et al. (2020) | 31 | 61.3% inpatients; 38.7% outpatients | 00:31 | 13.9 (4.25) | Anorexia nervosa | 1.8 yrs | NA | NR | France |
| Freeman et al. (2016) | 30 | 7% inpatients; 93% oupatients | VR cognitive therapy  = 10 :5; VR exposure = 6:9 | VR cognitive therapy = 42,1 (13,4); VR exposure = 40,6 (14,4) | Schizophrenia (20), schizoaffective disorder (3), Delusional disorder (2), or Psychosis Not Otherwise Specified(5) | NR | NR | NR | United Kingdom |
| Freeman et al. (2022) | 346 | 1% inpatients; 99% outpatients | 231:111 other: 1; prefer not to say 2; missing: 1 | **gameChange VR therapy + TAU**: 36.6 (12.8) **TAU:** 37.8 (12.2) | Schizophrenia (138), Schizotypal disorder (3), Delusional disorder (4), Brief psychotic disorders (14), Schizoaffective disorder (26), Other psychotic disorder (5), Unspecified psychosis (116), Bipolar disorder with psychotic features (8), Depressive disorders with psychotic features (20), Major depressive disorder with psychotic features (6) | NR | Mixed | NR | United Kingdom |
| Freeman et al. (2023) | 122 | 3.3% inpatients; 96.7% outpatients | 39:83 | 37.3 (13.2) | Schizophrenia (52), Schizotypal disorder (1), Delusional disorder (2), Brief psychotic disorder (3), Schizoaffective disorder (12), Other psychotic disorder (2), Unspecified psychosis (40); Bipolar disorder with psychotic features (2); Depressive disorders with psychotic features (5), Major depressive disorder with psychotic features (3) | NR | Yes (107), No (9), NR (1) | NR | United Kingdom |
| Giguère et al. (2023) | 19 | NR | 15:04 | 39.4 (9.86) | Cannabis use disorder (19); Other substance use disorders (19); Psychotic disorder (16); Bipolar disorder (1); Major depressive disorder (2); Antisocial personality disorder (8); Borderline personality disorder (6) | NR | NR | NR | Canada |
| Giguère et al. (2024) | 32 | NR | 27:05 | 36.6 (9.47) | Cannabis use disorder (32); Other substance use disorders (32); Psychotic disorder (25); Bipolar disorder (2); Major depressive disorder (5); Antisocial personality disorder (9); Borderline personality disorder (7) | NR | Yes | NR | Canada |
| Hesse et al. (2017) | 21 | NR | 15:06 | 34.52 (8.69) | Schizophrenia or schizoaffective disorder | NR | Yes (stable ≥ 1 week or unmedicated; no benzodiazepines ≥ 3 days before VR) | NR | Germany |
| Huang et al. (2021) | 32 | NR | 16:16 | 42.69 (9.01) | Schizophrenia (remission stage) | 18.2 yrs | Yes | NR | China |
| Just et al. (2024) | 30 | 37% inpatients; 63% day hospital | 14:16 | 69.47 (8.82) | Organic mental disorder (6), Alzheimer's dementia (4), Substance abuse disorder (1), Schizophrenia (3), Mood disorders (17) | NR | NR | NR | Germany |
| Kramer et al. (2013) | 14 | NR | 12:02 | 32.9 (7.8) | Post-traumatic stress disorder | NR | NR | NR | United States |
| Nijman et al. (2023) | 81 | 4% inpatients; 93% outpatients (remaining 2 participants = incomplete data) | 56:25 | 37.8 (11.4) | Schizophrenia (48), Schizoaffective disorder (18), Brief psychotic disorder (1), Schizophreniform disorder (1), Delusional disorder (1), Other psychotic disorder (11) | 11 yrs (8.8) | Yes | NR | Netherlands |
| Nijman et al. (2020) | 22 | 14% inpatients; 86% outpatients | 16:06 | 35.95 (11.68) | Schizophrenia (17);  Schizoaffective disorder (2); Psychotic disorder, not otherwise specified (3) | 13.22 yrs (11.22) | Yes | NR | Netherlands |
| Provenzano et al. (2024) | 26 | 100% day hospital patients | 00:26 | 20,61 (3,6) | Anorexia nervosa (restricting type) | NR | NR | NR | Italy |
| Schroeder et al. (2024) | 60 | 30% inpatients; 70% outpatients | 00:60 | 19.1 (4.88) | Anorexia nervosa-restrictive type | NR | NR | NR | Italy |
| Overall sample | 1 255 | 7.7% inpatients; 4% day hospital; 88.3% outpatients | 716 men 535 women 1 other 2 prefer not to say 1 missing | 40.85 years (SD = 10.90) | Schizophrenia spectrum/psychotic disorders: 544 (43%); Mood disorders (bipolar/depression): 71 (6%); Eating disorders: 117 (9%); Substance use disorders: 103 (8%); Anxiety/PTSD: 14 (1%); Other: 406 (32%) | 11.06 (10.01) years | Yes = 18 (78%) No = 4 (17%),  Mixed/Partial = 2 (9%) | 17.9 – 39.4 | NA |

NR = Not reported; NA = Not applicable

**Table 2. Summary of results of mixed in- and outpatient studies**

| **Study** | **VR application** | **Nature** | **Purpose** | **Control group** | **Summary of Results** | **Effect size** |
| --- | --- | --- | --- | --- | --- | --- |
| **Schizophrenia and related psychotic disorders** | | | | | | |
| Altunkaya et al. (2022) | Game Change  gameChange | CBT and exposure Therapy | Relearn safety by repeated behavioural experiments in graded social VR situations | Yes (TAU) | VR + TAU did not improve health-related quality of life vs TAU alone. | NR |
| Da Costa & de Carvalho (2004) | Integrated Virtual Environment for Cognitive Rehabilitation | Cognitive training | Investigate patient acceptance and motivation in usingVR for cognitive rehabilitation of schizophrenia | No | Participants showed high motivation, successfully completed tasks, and experienced no adverse effects. VR was well accepted. | NR |
| Dietrichkeit et al. (2020) | Virtual reality and recognition task | Assessment | Evaluate feasibility/efficacy of VR training for improving social functioning and to assess social cognitive biases in schizophrenia | Yes (non-clinical controls) | People with schizophrenia showed higher confidence in incorrect responses (especially emotional recognition) and poorer facial recognition vs. Controls. Feedback did not improve cognitive insight. | **Confidence incorrect in emotional recognition :** d = .334 (miss);  d = .840 (false memory) **Confidence correct in recognition of faces** : d = .512 |
| Freeman et al. (2016) | VR cognitive therapy or VR exposure | CBT and exposure therapy | Reduce conviction and distress in persecutory delusions by testing threat beliefs and modifying safety behaviours within VR social situations | No | VR cognitive therapy reducing safety behaviours led to greater decreases in delusional conviction and distress than VR exposure, with improvements transferring to real-life situations than VR exposure with safety behaviours maintained. | **Ratings of the delusion**: d = 1.3 **Ratings in the behaviour test**: d = 0.8 |
| Freeman et al. (2022) | gameChange | CBT and exposure therapy | Reduce agoraphobic avoidance/distress in everyday situations in psychosis | Yes (TAU) | VR+TAU significantly reduced agoraphobic avoidance and distress compared with TAU alone, especially in those with severe avoidance at baseline. | **VR x TAU (Overall):** Agoraphobic avoidance: d = -0.18 Distress: d = -0.26 **VR x TAU (Severe Baseline avoidance)**: Agoraphobic avoidance: d = 0.68 Distress: d = 0.43 |
| Freeman et al.(2023) | gameChange | CBT and exposure therapy | Reduce anxiety in everyday social situations for patients with psychosis, Assess side effects of VR | No | Most participants were satisfied with VR therapy and reported positive experiences. Side effects included difficulties concentrating when wearing the headset, feelings of panic using VR, and worries following VR. Side effects did not limit therapeutic gains. | NR |
| Hesse et al. (2017) | Custom office-based VR scenario | Assessment | Assess feasibility and psychosocial effects of a VR-based social interaction task in psychosis, focusing on how social rejection influences paranoid ideation and working memory. | Yes (non-clinical controls) | Virtual social rejection increases paranoid ideation in people with schizophrenia; no significant effect on presence or working memory; barriers included simulator sickness and stress during sessions. | **Paranoid ideation** : n_p_^2^ = 0.17 **Presence** : d = 0.65 **Working memory**: n_p_^2^ = 0.007 (D-prime task) ;  n_p_^2^ = 0.013 (false alarms task) |
| Huang et al. (2021) | VR cognition training system(VRCTS) | Assessment | Evaluate cognitive function and test the reliability of VR in distinguishing remission-stage SZs from healthy controls. | Yes (non-clinical controls) | VRCTS completion times were significantly longer for SZs than controls; Outcomes correlated with MCCB scores, and the VRCTS distinguished SZs from controls with high accuracy, sensitivity, and specificity. | NR |
| Nijman et al. (2020) | Dynamic Interactive Social Cognition Training in Virtual Reality (DiSCoVR) | Cognitive training | Facial affect recognition, social perception, Theory of Mind and application of social cognition in social interactions | No | Significant improvement of emotional perception. | **Ekman 60 Faces Test**: d = -0.67 **BLERT**: d = 0.03 **TASIT-I**: d = -0.15 |
| Nijman et al. (2023) | DiSCovVR | Cognitive training | Facial affect recognition, social perception, Theory of Mind and application of social cognition in social interactions | Yes (active) | Results indicate an absence of treatment effects. | NR |
| **Eating and food intake disorders** | | | | | | |
| Fisher et al. (2020) | C2CARE PSY | Assessment | Comparison of body imagedisturbance evaluation in anorexia nervosa using VR-based avatars versus paper-based figure ratingscale | Yes (clinical) | No significant difference between VR and paper-based assessments; both methods correlated with psychometric parameters and showed that participants overestimated body size and preferred thinner figures. | NR |
| Provenzano et al. (2024) | Full Body Illusion task | Assessment | The app aimed to assess whether a Full Body Illusion (FBI) using under and normal-weight avatars’ bodies affects perceptual body image and body schema estimations in both individuals with anorexia nervosa and healthy controls. | Yes (non-clinical controls) | Participants with AN showed an increase of the body size overestimation following the embodiment of the normal-weight avatar, patients rated the normal-weight avatar as the most similar to themselves yet simultaneously deemed it the least attractive and the most repulsive, and all participants reported feeling thinner than usual after embodying the underweight avatar | **Body size overestimation**: η^2^ = 0.10 **Self-similarity** : η^2^ = 0.509 **Implicit disgust** : η^2^ = 0.214 **Feelings of** **thinness/fatness** : η^2^ = 0.112 **Attractiveness** : η^2^ = 0.292 |
| Schroeder et al. (2024) | Kinematic task in virtual reality | Assessment | Distinguish between continuous avoidance of food stimuli and inhibitory control over food-related actions. | Yes (non-clinical controls) | Differences in reaction times and food preferences based on the presence or absence of an eating disorder. | **Reaction time food**: d = 0.82 **Food liking [VAS]**: d = 0.85 **Food wanting [VAS]**: d=1.22 |
| **Post-traumatic stress disorder** | | | | | | |
| Kramer et al. (2013) | Custom software developed at Virtual Reality Medical Centers, San Diego | Assessment | Explore acceptability, appropriateness, and effectivenessof VR as a PTSD assessment tool | No | Veterans were able to immerse themselves in VR, experiencing memories and physiological responses, Iraq-related thoughts/behaviours, and were positive about VR; concerns about potential negative reactions. | NR |
| **Mixed samples combining multiple diagnoses** | | | | | | |
| Giguère et al. (2023) | Unity 3D | CBT and exposure therapy | Reduce cannabis use and improve related psychosocial outcomes in people with severe mental disorders | No | Significant reductions in cannabis use, increased motivation for change, and no increase in use of other substances; quality of life trended upward, and psychiatric symptoms remained stable. | **Frequency of cannabis use**: d= 0.313 **Motivation for change**: d = 0.523 **Use of other substances**: d = 0.397 **Quality of life**: d = 0.220 |
| Giguère et al. (2024) | Unity 3D | CBT and exposure therapy | Reduce cannabis use and improve coping skills, psychiatric symptoms, and quality of life in individuals with CUD and serious mental disorders. | No | Significant reductions in cannabis use maintained up to 12 months; improvement of quality of life, coping strategies, and psychiatric symptoms; no increase in use of other substances. | **Consumed cannabis over time (12-month follow-up visit)**: d = 0.804 **Quality of life**: d = 0.675 (3-month follow-up); d = 0.532 (12-month follow-up) **Coping strategies**: d = 0.769 **Psychiatric symptomatology**: d = 0.782 |
| Just et al. (2024 | Nature Treks VR | Assessment | This explorative study examined the feasibility of using Virtual Reality (VR) for elderly patients with psychiatric illness, focusing on the sense of presence as the primary outcome | No | Notable sense of presence in the virtual environment, particularly in terms of spatial presence. Motion sickness was reported by a minority of patients. The average well-being during the VR experience was reported as high. Sense of presence and motion sickness showed a significant negative correlation. Presence, motion sickness and well-being were not significantly correlated with age, nor did they differ significantly between groups | NR |

Notes: TAU = Treatment as usual; NR = non-reported; Dynamic Interactive Social Cognition Training in Virtual Reality (DiSCoVR); VR = Virtual reality; CBT = Cognitive Behavioral Therapy; CUD = Cannabis Use Disorder; MCCB = MATRICS Consensus Cognitive Battery; SZ = Schizophrenia;. D-prime = ratio between the hit rate and the false alarms, BLERT = Bell-Lysaker Emotion Recognition Test, TASIT-I = The Awareness of Social Inference Task (assessing emotion perception), TMT-A and B = The Trail Making Test with numbers (A) or numbers and letters (B), VAS = Visual analog scale

**Supplement 5: Softwares and Hardwares**

The resources required for implementation were documented in over 20 studies, encompassing both human and material components. Across studies, various VR systems and protocols were employed, typically involving head-mounted displays (e.g., *HTC Vive*, *Meta Quest*, *Samsung Gear VR*, *Oculus Rift*), joysticks, tracking sensors, and supplementary tools such as gaze control or 360° video.

Some interventions used customized software applications tailored to therapeutic objectives, including *VREDIM* (Riva, 2003), *Z301* (Freeman, 2022, 2023), *VRelax* (Nijman, 2023), and *NHGRI’s* “virtual buffet” (Faulkner, 2024). Others relied on open-source or research-specific platforms (e.g., NeuroVR in Cesa, 2013). In several cases, the virtual environments were developed using 3D modelling and game engine tools such as *Unity3D®*, *MakeHuman*, *3dsMax*, or *Adobe Photoshop* (e.g., Provenzano, 2020), which supported the design and rendering of the immersive settings used in interventions
